# Comparison of fungal communities and nonvolatile flavor components in black *Huangjiu* formed using different inoculation fermentation methods

**DOI:** 10.3389/fmicb.2022.955825

**Published:** 2022-07-22

**Authors:** Pingping Li, Rui Su, Qi Wang, Kunyi Liu, Hai Yang, Wei Du, Zhengang Li, Song Chen, Bin Xu, Wen Yang

**Affiliations:** ^1^Sericulture and Apiculture Research Institute, Yunnan Academy of Agricultural Sciences, Mengzi, China; ^2^College of Wuliangye Technology and Food Engineering, Yibin Vocational and Technical College, Yibin, China; ^3^Luzhou Greenland Wine Co., Ltd., Luzhou, China

**Keywords:** black Huangjiu, sequential inoculation, fungal communities, non-volatile components, correlation analysis

## Abstract

Black *Huangjiu* (BH) is a traditional alcoholic beverage in China, which is very popular among people. The different methods (simultaneous inoculation, sequential inoculation), were applied to ferment BH in this study, which were investigated the changes in the composition of fungal communities and non-volatile flavor compounds (nVFCs) by high-throughput sequencing (HTS) and ultraperformance liquid chromatography–tandem mass spectrometer (UPLC MS/MS). The results showed that *Rhizopus* and *Saccharomyces* were the predominant fungal genera throughout fermentation, and 471 nVFCs were detected in BH after fermentation. Compared to that observed simultaneous inoculation, *Rhizopus* increased at the end of sequential fermentation, and the contents of the organic acids and their derivatives increased significantly [variable importance in the projection (VIP) > 1.0, *p* < 0.05, fold change (FC) > 2], while that of lipids and lipid-like molecules decreased significantly (VIP > 1.0, *p* < 0.05, FC < 0.5). Through the correlation analysis of 32 nVFCs with significant differences (VIP > 1.0, *p* < 0.05, FC >32 or < 0.03) and the community, it was found that lipids and lipid-like molecules (12) and organic acids and their derivatives (10) were significantly (*p* < 0.05) negatively correlated with *Saccharomyces*, but they were significantly (*p* < 0.05) positively correlated with *Rhizopus*. Compared with simultaneous inoculation, BH fermented by sequential inoculation, the taste was stronger, sweeter, mellow, and softer. Our findings provide information on nVFC dynamics and will aid in the selection of beneficial strains to improve BH quality.

## Introduction

Black *Huangjiu* (BH) is a traditional alcoholic beverage and usually fermented with black glutinous rice as the main raw material, which is full of nutrients and may thus be beneficial to people. As a traditional *Huangjiu*, it is deeply loved by consumers and has been popular in China for thousands of years due to its unique flavor, healthcare effects, and potential medicinal properties, including antioxidant, hypoglycemic, lipid-lowering, hypotensive, and renal protective activities (Jiang et al., 2020; Yang et al., 2020; Chen et al., 2021). It is well known that the flavor characteristics of BH refer to aroma, taste, and color, which mainly depend on the differences of raw materials, starter, technique, and microbial diversity in the traditional brewing process (Ren et al., 2019, 2020).

The traditional brewing process of BH involves inoculation of starter culture in the materials, saccharification in an open environment, and subsequent aging for fermentation (Jiang et al., 2020; Chen et al., 2021). Moreover, in this complex environment, a series of changes occur in the microbial flora, which affect the unique flavor and color of Huangjiu (Carrau et al., 2015). Numerous studies have documented that the composition of microbial communities directly affects the metabolic functions of microorganisms and the production of flavor compounds (Huang et al., 2018; Liu et al., 2019; Park et al., 2019). Thus, altering the proportion of particular microbial may be help for improving the quality of fermented foods in some but not all systems.

The starter is of central importance for brewing BH, which determines the microbial community and the primary components of enzymes therein (Chen et al., 2021). In general, the taste of BH is often related to some autolysate of *Lactobacillus* (Xie et al., 2007) and array of compounds, including sugars, organic acids, amino acids, and peptides, produced by enzymatic biochemical reactions and microbial metabolism (Yang et al., 2020; Chen et al., 2021). Recently, researchers investigated BH samples from different years by liquid chromatography, sensory evaluation, and electronic tongue analysis (Chen et al., 2021). Rang et al. (2016) found that ethanol and higher alcohols were generated by the metabolizing of yeast. In recent years, the biosynthetic pathways and related regulatory genes of flavor substances in BH, such as higher alcohols, acetates, and fatty acid ethyl esters, has been reported (Chen et al., 2021). Currently, it was concluded that the flavor characteristics of BH have been well studied and still be focused on volatile components, such as alcohols, aldehydes, and volatile acids. In fact, there are a small quantity of non-volatile compounds in BH. However, the general functions of these compounds are not yet fully understood and whether they have a significant impact on the flavor features of BH. Therefore, it is necessary to investigate the change of non-volatile flavor compounds and their relationships with the microbial community during fermentation to improve the quality of BH.

Black glutinous rice (*Oryza sativa* L.), one of the most popular varieties of brown rice, is chosen as a traditional raw material of producing BH and known as the “black pearl” because of high content of phenols, flavonoids, and especially anthocyanins (Shen et al., 2009). In recent years, some literature have been reported special flavor BH, which were produced by black glutinous rice and other raw materials (Jiang et al., 2020; Zhao et al., 2020). The fruits of mulberry (*Morus alba* L.) plants are not only edible and delicious (Sanchez-Salcedo et al., 2016), but also full of antioxidants and various other nutritious biological ingredients that are very beneficial to human health, including vitamins, polyphenols, flavonols, phenolic acids, and anthocyanins (Yuan and Zhao, 2017; You et al., 2018). If black glutinous rice and mulberry were taken as raw fermentation materials for producing BH, then it may be featured of unique fragrance, taste and color, which meets the growing demand of consumers for healthy products. But it is a pity that no research has been reported on BH fermented using black glutinous rice and mulberry up to now.

In recent years, microbiota research has attracted widespread attention based on high-throughput sequencing (HTS) technology, which is attributed to low cost, more sequencing depth, fast, and accurate assessment of complex microbial taxa (Mayo et al., 2014; Lee et al., 2017). Currently, this technology is extensively applied for comprehensive analysis of microorganism in various fermented foods, such as Shaoxing *Huangjiu* (Nie et al., 2015), black glutinous *Huangjiu* (Zhao et al., 2020), and *Huangjiu* (Tian et al., 2022). In the study, the dynamical fungal community structures were evaluated during BH fermentation by HTS. Meanwhile, a metabolic map was established based on sequential inoculation and traditional simultaneous inoculation to clarify the differential metabolites and their correlation with microorganism. The work may shed light on the comprehensive insights regarding of microbial interactions and dynamic changes of nVFCs during BH fermentation and provide new ideas for producing BH.

## Materials and methods

### Materials

Fresh mulberries (Daoshi, *Morus atropurpurea* Roxb.) were obtained from the Yunnan Academy of Agricultural Sciences (Yunnan, China) in March 2021, and black glutinous rice (the starch content was 71.26%) was procured from Shenyang Xinchang Grain Trading Co., Ltd. (Liaoning, China). The dried active *Saccharomyces cerevisiae* and liqueur koji (rice flour fermented using *Rhizopus oryzae*) were purchased from Angel Yeast Co., Ltd. (Hubei, China).

### Test flow of BH

The operation points of test flow were carried out as follows: removing impurities and stems from fresh mulberries, adding distilled water of equal quality to the mulberries after washing with distilled water, squeezing the mulberries using a MJ-WJE2802D juicer (Midea Group Co., Ltd., China), and filtering by four layers of gauze to obtain the mulberry juice. After soaking for 12 h with the distilled water, the black glutinous rice was steamed for 45 min until fully cooked, followed by the addition of mulberry juice of equal quality, and cooled to 30°C. Meanwhile, the mixtures were inoculated with 0.5% liqueur koji to obtain SF1, and saccharification was performed at 28°C for 24 h. Then, 0.1% yeast suspension was activated using distilled water at 30°C and added to the mixtures (mark as SF2), which were fermented under static conditions at 25°C for 48 h and labeled as SF3. Fermentation procedure of control (CK) was performed similar to that of SF, except that the microorganisms used for inoculation were different. For CK1, the microorganisms inoculated in the mixtures were 0.5% liqueur koji and 0.1% yeast suspension at the beginning of fermentation. Thereafter, CK1 was go on static fermentation at 28°C for 24 h and followed by sequential fermentation at 25°C for 48 h to harvest samples, named CK2 and CK3, respectively. These fermented samples were taken repeatedly three times and conserved at −80°C until required.

### Sensory evaluation

The sensory characteristics of BH were examined by a sensory evaluation panel consisting of 11 (6 men and 5 women) wine-tasters according to the Chinese standard (GB/T 13662-2018) and (Shen et al., 2021). The four aspects evaluated were appearance (0–10 score), aroma (0–30 score), taste (0–40 score), and typicalness (0–20 score; Table 1). A total sensory score was calculated from the four individual scores.

**Table 1:** Sensory score standards of BH.

	The weight of sensory index	Grading standard
Appearance	0.1	Hull of glossy, no obvious suspension
Aroma	0.3	Rich and fruity, no undesirable aroma
Taste	0.4	Harmonious, delicate, mellow, and full-bodied
Typicalness	0.2	Unique style of *Huangjiu* and elegant quality

Bad very much: 0–19 score; Bad moderately: 20–39 score; Bad: 40–59 score; Good: 60–79 score; Good moderately: 80–89 score; Good very much: 90–100 score.

### Illumina MiSeq sequencing

The fungal genomic DNA was extracted, respectively, from the above 18 BH fermented samples to analyze their taxonomic composition of fungal communities. The quality of DNA was monitored by 1% agarose gel electrophoresis. DNA concentration and purity were determined by Nanodrop2000. ITS genes of distinct regions (ITS3/ITS4) were amplified used the universal primer ITS3F (5′-GCATCGATGAAGACGCAGC-3′) and ITS4R (5′-TCCTCGCTTATTGATGC-3′) with the barcode by PCR (ABI GeneAmp® 9700). The PCR mixtures contain 2 × Pro Taq Mix 4 μL, forward primer (5 μmol/l) 0.8 μL, reverse primer (5 μmol/l) 0.8 μL, template DNA 10 ng, and finally ddH2O up to 20 μL. Thermal cycling consisted of initial denaturation at 95°C for 3 min, followed by 35 cycles of denaturation at 95°C for 30 s, annealing at 55°C for 30 s, and elongation at 72°C for 45 s, then finally at 72°C for 10 min. Each sample was repeated three times. These works, including PCR product quantification, qualification, and library preparation, were done by the company (Shanghai Meiji Biomedical Technology Co., Ltd., Shanghai, China). At last, the library was sequenced on an Illumina MiSeq platform and 300 bp paired-end reads were generated.

### Processing of sequencing data

The offline data were assigned to samples based on their unique barcode and then removed the barcode and primer sequence. According to the overlapped sequence between reads, the paired reads were merged using FLASH software (version 1.2.11; Magoč and Salzberg, 2011), and the splicing sequences were raw data. Afterward, the raw data were quality filtered by FASTP software (version 0.20.0)^1^ (Chen et al., 2018) and chimera sequence was removed to gain an effective sequence for subsequent analysis. Effective sequences of all samples were clustered according to 97% identify and divided into different operational taxonomic units (OTUs) by Uparse (version 7.0.1090; (Edgar, 2013).^2^ Each OTU representative sequence was analyzed and annotated by RDP Classifier based on the Bayesian algorithm of QIIME (version 2.11)^3^ and the Unite fungal database (version 8.0)^4^ with a confidence threshold of 0.7. Moreover, the composition and richness of community were counted at each taxonomic level.

### nVFC analysis

In this study, SF3 and CK3 were selected and delivered to the company for determination the nVFCs of samples by ultra-high performance liquid chromatography–tandem mass spectrometry (UPLC-MS/MS) analysis (Shanghai Meiji Biomedical Technology Co., Ltd., Shanghai, China). The following works were accomplished according to our recent study, which included the extraction of nVFCs, UPLC separation, ESI-MS/MS monitoring, and data processing (Liu et al., 2020). Identification of nVFC was dependent on the primary and secondary MS data, annotated against an in-house database, or the matches of both retention times and MS data to some standard compounds in Metware. According to the Metabolomics Standards Initiative (MSI), these methods achieved level-2 and level-1 annotations, respectively. Principal co-ordinate analysis (PCoA) and orthogonal partial least-squares-discriminant analysis (OPLS-DA) were applied to compare the nVFC of groups by MetaboAnalyst 5.0 (Pang et al., 2021).^5^ In the OPLS-DA model, the overall contribution of each variable was ranked by variable importance in the projection (VIP). Therein, nVFCs were considered differentially changed when variables with VIP > 1.0, *p* < 0.05 (Student’s *t*-test), and fold change (FC) > 2 or < 0.5.

### Statistical analysis

Data were standardized for alpha diversity analyzation with abundance-based coverage estimator (ACE), Chao, Shannon, and Simpson indexes of each sample, so that reflected the richness and evenness of microbial community. Principal co-ordinate analysis (PCoA) was applied based on weighted UniFrac distance to compare the fungal community composition at different fermentation stages of all samples. Mann–Whitney test was taken to analyze the significance of alpha diversity index of fungal communities between different samples. Pearson’s correlation analysis and cluster analysis (data were normalized) were performed to assess the relationship between microbe and nVFCs after BH fermentation using R Project 3.6.1.

### Data availability statement

The amplicon data of fungal ITS are available at BioProject^6^ with the accession number of PRJNA846490.

## Results

### Fungal diversity during the fermentation of BH

A total of 891,749 high-quality sequences were obtained from all samples and their sequences length were average 365 bp. These effective sequences were finally clustered into 21 and 13 OTUs for SF and CK group, respectively, according to 97% identity. Each sample of control group was assigned to 11, 6, and 4 OTUs for CK1, CK2, and CK3, respectively, while trial group were that 19, 6, and 3 OTUs for SF1, SF2, and SF3 (Supplementary Figure S1). Additionally, the two groups shared two different OTUs as showed in the Venn diagram (Supplementary Figure S1). The rarefaction curve inclined to the saturation platform as well as the effective sequences was more than 99% for per sample, indicating that the sequencing depth has nearly covered all species in the sample.

**Figure 1:**
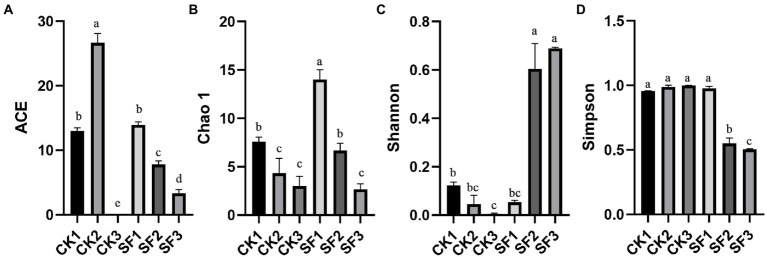
Comparative analysis of α-diversity indexes of fungal flora in BH at different fermentation stages.

Alpha diversity analysis (Figure 1) showed that the index of ACE (Figure 1A) and Chao1 (Figure 1B) of SF1 from the trial group was significantly higher than those of SF2 and SF3 (*p* < 0.05), indicating that the community richness of samples unfermented in the treatment groups was significantly higher than that of samples fermented for 24 h and 72 h. The ACE index (Figure 1A) of CK2 was significantly higher than that of CK3 (*p* < 0.05). Although there was no significant difference in Chao1 index (Figure 1B) (*p* > 0.05), the overall trend was downward in the matter of colony richness of the control group at the two different fermentation stages. Judging from the Simpson index, (Figure 1D) a significant increase was displayed in the fungi diversity of SF group as prolongation of fermentation (*p* < 0.05), and a similar trend was found in this group for Shannon index (Figure 1C). However, Simpson index (Figure 1D) had no significant difference in the control group (*p* > 0.05), suggesting that the fungal diversity in the control group did not change significantly with the progress of fermentation. In a word, the results of fungal colony diversity were consistent with the OTU analysis among all samples of BH.

**Figure 2:**
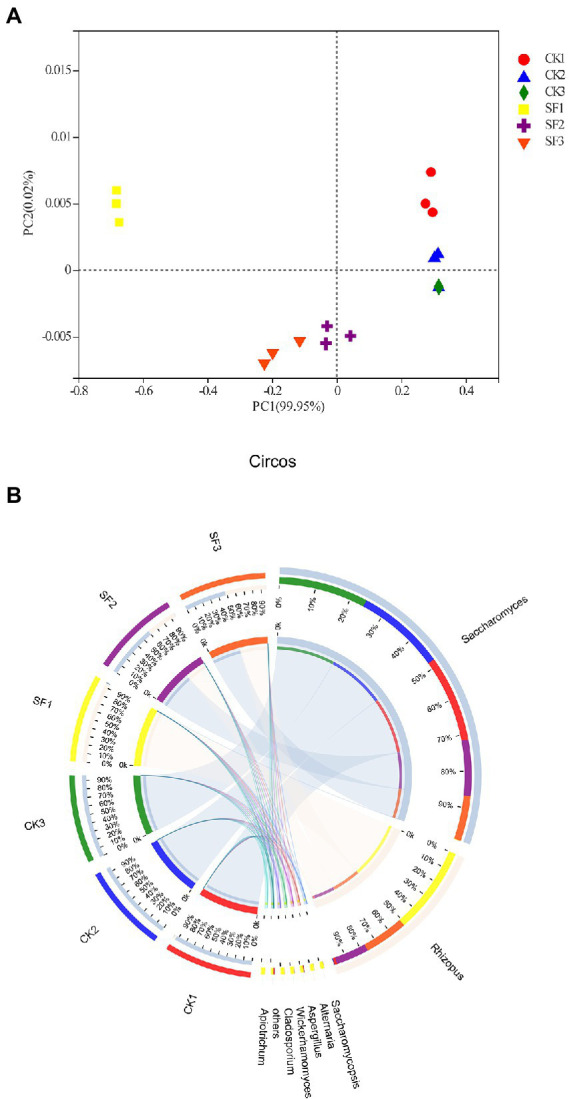
Principal co-ordinate analysis based on weighted distance **(A)** and fungal communities in fermented samples at the genus level **(B)**.

Here, the results of PCoA analysis showed that there were significant differences in fungal community structure between CK group and SF group at the same fermentation stage (*p* < 0.05; Figure 2A). The distribution of the CK1 group that has not been fermented were far away from those of CK2 and CK3 groups that have been fermented, indicating that the fungal community structure of CK1 group was significantly different from that of CK2 and CK3 groups (*p* < 0.05) in the genus level. However, the close distance between CK2 and CK3 demonstrated that their community structure was similarity, and alike rule was also found in the experimental group. But as a consequences of more different community structure, the distribution of SF1 was farther away from the SF2 and SF3. Overall, these works proved that the different inoculation ways could significantly affect the composition of the fungal community (*p* < 0.05).

### Changes in fungal dynamics during fermentation

According to the result of species annotation, all OTUs of samples were classified into 4 phyla, 9 classes, 11 orders, 16 families, 20 genera, and 24 species. Among of which, eight fungal genera were found that their relative abundance was greater than 0.0001% (Figure 2B). In the control group (CK1, CK2, and CK3), *Saccharomyces* was absolutely dominant due to an average richness 97.60%. For the same stage samples which have not yet activated fermentation, *Rhizopus* accounted for 3.0% in the CK1, while it was the preponderant strain in SF1 group with a very large proportion of 99.0% (Figure 2B). The sample of CK2 group was harvested after CK1 being fermented for 24 h, and then the relative abundance of *Saccharomyces* increased to 99%, but yet that of *Rhizopus* decreased to 0.60% therein. However, *Saccharomyces* (63.0%) and *Rhizopus* (37.0%) were the main fungi in SF2 sample, which was obtained by inoculating *saccharomyces* into SF1 saccharified for 24 h. Subsequently, final samples of control or trial group were acquired (namely CK3, SF3) after 48 h of fermentation. Of which, the dominant fungi of CK3 were *Saccharomyces* (99.95%) and a small amount of *Rhizopus* (0.05%), while that of SF3 were equal *Saccharomyces* and *Rhizopus* with relative abundance occupying for 50.0%, respectively. In brief, the preponderant fungus in the sample of control group has always been *Saccharomyces* and *Rhizopus* was slowly decreased from 3 to 0.05% during the two different fermentation stages (24 h and 48 h), whereas that of yeast decreased by 20.63% and *Rhizopus* increased by 35.14% in the experimental group after inoculating with yeast (SF2).

### Sensory evaluation

**Figure 3:**
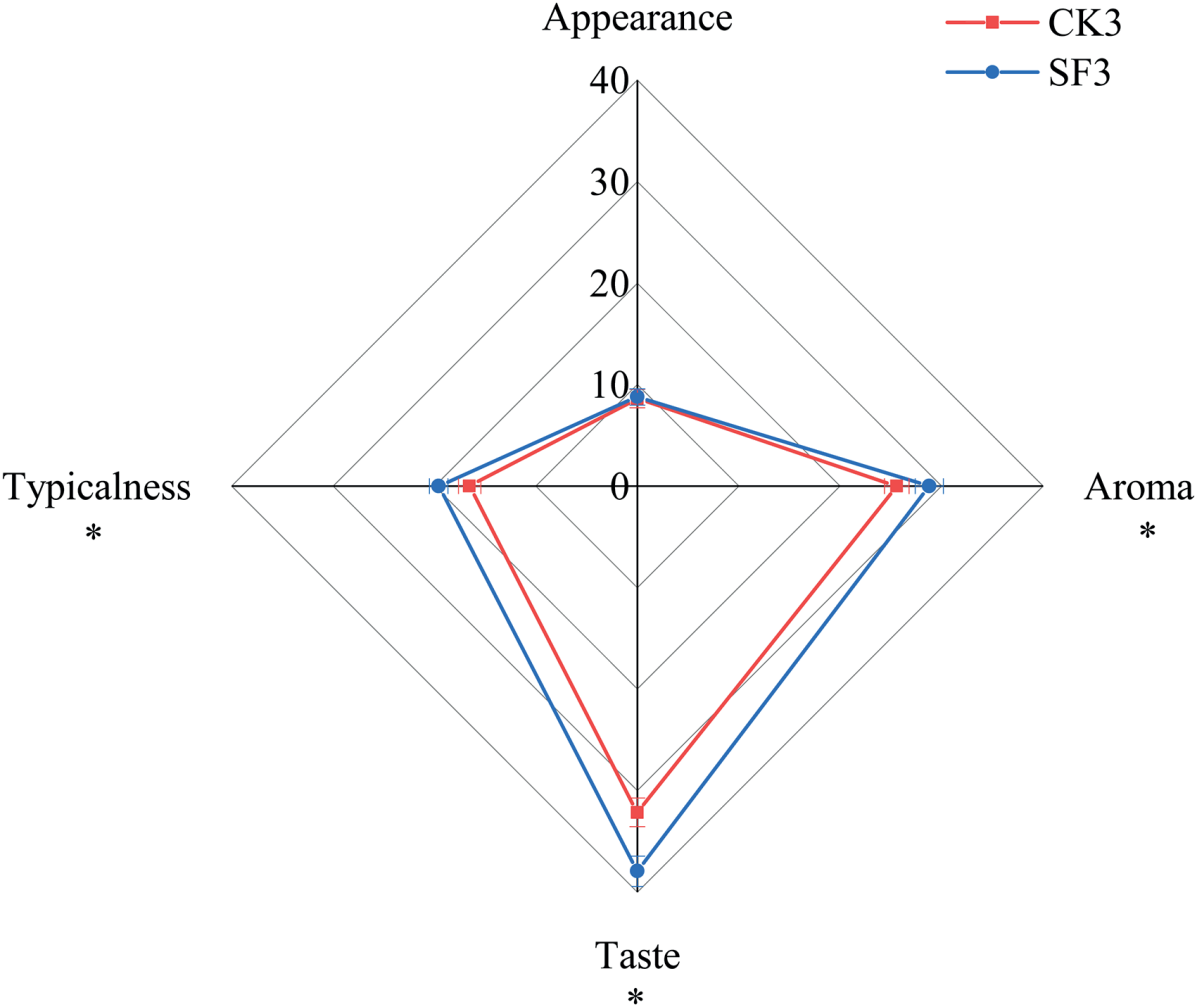
The sensory evaluation results of BH samples in CK3 and SF3 group. ^*^p < 0.05.

The sensory characteristics of the BH samples were described by the 11 sensory panelists. The radar map of the mean sensory scores of the BH samples sourced from CK3 and SF3 is shown in Figure 3. The sensory score of SF3 (95.07 ± 1.56) was significantly higher (*p* < 0.05) than that of CK3 (82.89 ± 1.13), while the scores of aroma (28.77 ± 1.06), taste (37.94 ± 1.42), and typicalness (19.58 ± 1.24) of SF3 were significantly higher (*p* < 0.05) than that of CK3. SF3 had brown purple color; the *Huangjiu* special aroma (full bodied and mellow aroma); sweet, mellow, soft, and refreshing taste; and the wine’s flavor was co-ordinated. CK3 had brown purple color; an aroma of *Huangjiu*, full bodied but not intense aroma; sweet, slightly mellow, and refreshing taste; and the wine’s flavor was generally co-ordinated.

### nVFCs of BH after fermentation

**Figure 4:**
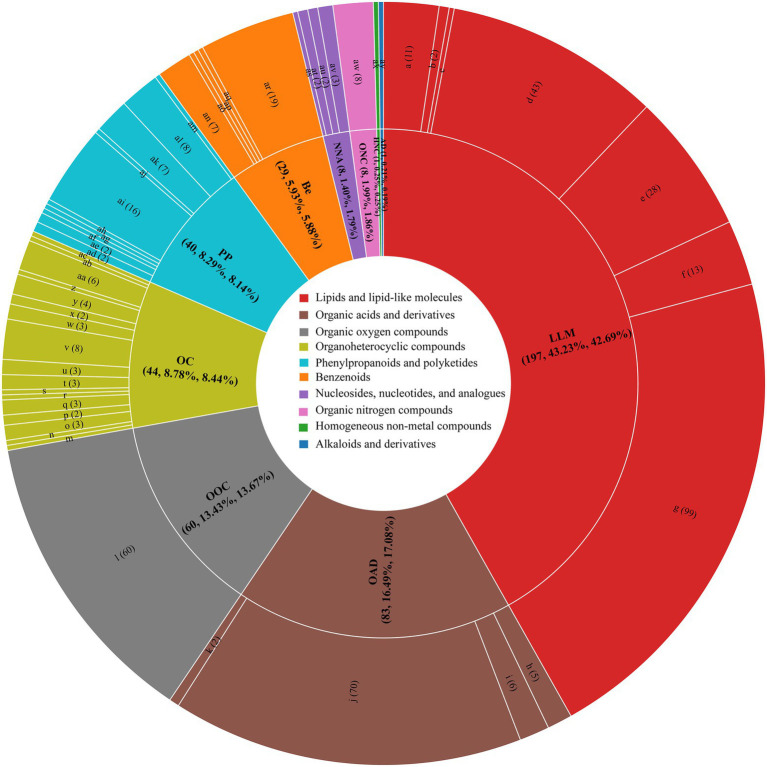
Sunburst of the contribution of nVFCs in SF3 and CK3 in OPLS-DA. a, steroids and steroid derivatives; b, sphingolipids; c, saccharolipids; d, prenol lipids; e, glycerophospholipids; f, glycerolipids; g, fatty Acyls; H, keto acids and derivatives; i, hydroxy acids and derivatives; j, carboxylic acids and derivatives; k, carboximidic acids and derivatives; l, Organooxygen compounds; m, pyrrolidines; n, pyrroles; o, pyridines and derivatives; p, pyrans; q, pteridines and derivatives; r, oxepanes; s, naphthofurans; t, lactones; u, indoles and derivatives; v, imidazopyrimidines; w, heteroaromatic compounds; x, dihydrofurans; y, diazines; z, benzotriazoles; aa, benzopyrans; ab, benzodioxoles; ac, azolidines; ad, stilbenes; ae, phenylpropanoic acids; af, macrolides and analogs; ag, Kavalactones; ah, isoflavonoids; ai, flavonoids; aj, diarylheptanoids; ak, coumarins and derivatives; al, cinnamic acids and derivatives; am, 2-arylbenzofuran flavonoids; an, phenols; ao, phenol ethers; ap, phenanthrenes and derivatives; aq, naphthalenes; ar, benzene and substituted derivatives; as, ribonucleoside 3′-phosphates; at, pyrimidine nucleotides; au, pyrimidine nucleosides; av., pyrimidine nucleosides; aw, organonitrogen compounds; ax, non-metal oxoanionic compounds; ay, morphinans.

A total of 471 nVFCs were detected in CK3 and SF3 as shown in Figure 4, including lipids and lipid-like molecules (197), organic acids and derivatives (83), organic oxygen compounds (60), organoheterocyclic compounds (44), phenylpropanoids and polyketides (40), benzenoids (29), organic nitrogen compounds (8), nucleosides, nucleotides, analogs (8), homogeneous nonmetal compounds (1), and alkaloids and derivatives (1). Among which, 213 differential nVFCs were found between CK3 and SF3 (Figure 4). Compared to CK3, the compound composition changed in SF3 group was significantly changed (*p* < 0.05), the relative proportion of lipids and lipid-like molecules decreased from 43.23 to 42.67%, and that of organic acids and their derivatives increased from 16.29 to 17.08% (Figure 4; Supplementary Table S1).

**Figure 5:**
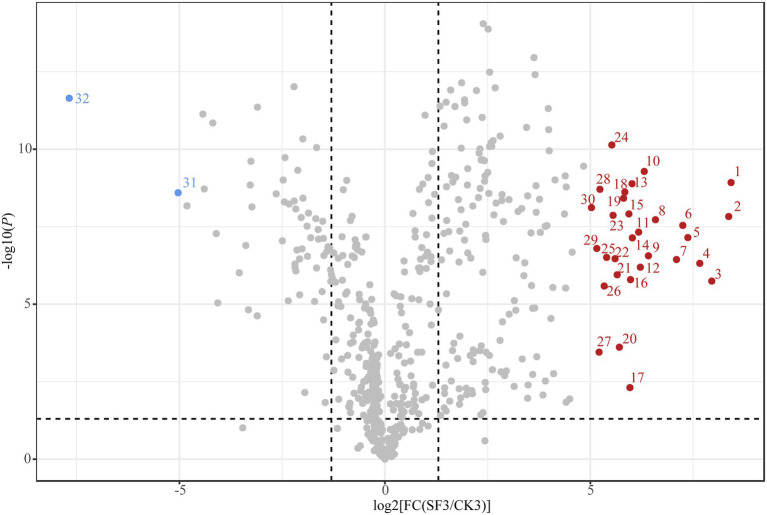
Volcano plot showing the contribution of nVFCs in SF3 and CK3 in OPLS-DA. The first data in the brackets is the number of metabolites, the second data are the percentage of each metabolites in the total nVFCs in CK3 and the third data is that in SF3. 1: Prolyl-Valine; 2:LysoPE(0,0/22,0); 3:Enkephaline, (D-Ala)2-Leu; 4: Agavoside A; 5: 6-{[(16S)-5,7-dihydroxy-8,8,12,16-tetramethyl-3-[1-(2-methyl-1,3-thiazol-4-yl)prop-1-en-2-yl]-10-methylidene-9-oxo-17-oxa-4-azabicyclo[14.1.0]heptadec-4-en-11-yl]oxy}-3,4,5-trihydroxyoxane-2-carboxylic acid; 6: Gamma-Glutamylphenylalanine; 7: 6-{[(16S)-5,7-dihydroxy-8,8,10,16-tetramethyl-3-[1-(2-methyl-1,3-thiazol-4-yl)prop-1-en-2-yl]-12-methylidene-9-oxo-17-oxa-4-azabicyclo[14.1.0]heptadec-4-en-11-yl]oxy}-3,4,5-trihydroxyoxane-2-carboxylic acid; 8: Asp-Phe; 9: Physagulin D; 10: (+/−)-Hexanoylcarnitine; 11: Isoleucyl-Leucine; 12: Adenosine 3′-monophosphate; 13: PS(MonoMe(11,5)/MonoMe(11,3)); 14: Uridine diphosphate-N-acetylglucosamine; 15: Physapruin B; 16: 7a-Hydroxy-5b-cholanic acid; 17: Isoleucyl-Isoleucine; 18: Isoleucyl-Tyrosine; 19: Digalacturonate; 20: 2,10-Bisaboladiene-1,4-diol; 21: (+/−)-Octanoylcarnitine; 22: Isoleucyl-Alanine; 23: 2-(2,4-dihydroxyphenyl)-5,7-dihydroxy-6-(3-methylbut-2-en-1-yl)-3,4-dihydro-2H-1-benzopyran-4-one; 24: N-(2-Phenylethyl)-acetamide; 25: L-cis-Cyclo(aspartylphenylalanyl); 26: Leucyl-Alanine; 27: Sulfolithocholylglycine; 28: Curcumadiol; 29: Caryoptosidic acid; 30: Ascorbyl stearate; 31: Acetylcholine; 32: 1-Methylhypoxanthine.

The detailed parameters of the nVFCs are listed in Supplementary Table S2. Overall, the level of 191 nVFCs in SF3 increased significantly (VIP > 1.0, *p* < 0.05, FC > 2) compared to those in CK3, of which that of 30 nVFCs increased more than 32-fold, including prolyl-valine, lysoPE(0:0/22:0), enkephalin, (D-Ala)2-Leu, agavoside A, 6-{[(16S)-5,7-dihydroxy-8,8,12,16-tetramethyl-3-[1-(2-methyl-1,3-thiazol-4-yl)prop-1-en-2-yl]-10-methylidene-9-oxo-17-oxa-4-azabicyclo[14.1.0]heptadec-4-en-11-yl]oxy}-3,4,5-trihydroxyoxane-2-carboxylic acid, gamma-Glutamylphenylalanine, 6-{[(16S)-5,7-dihydroxy-8,8,10,16-tetramethyl-3-[1-(2-methyl-1,3-thiazol-4-yl)prop-1-en-2-yl]-12-methylidene-9-oxo-17-oxa-4-azabicyclo[14.1.0]heptadec-4-en-11-yl]oxy}-3,4,5-trihydroxyoxane-2-carboxylic acid, asp-Phe, physagulin D, (+/−)-hexanoylcarnitine, isoleucyl-Leucine, adenosine 3′-monophosphate, PS(MonoMe(11,5)/MonoMe(11,3)), uridine diphosphate-N-acetylglucosamine, physapruin B, 7a-Hydroxy-5b-cholanic acid, isoleucyl-Isoleucine, isoleucyl-Tyrosine, digalacturonate, 2,10-bisaboladiene-1,4-diol; 21: (+/−)-Octanoylcarnitine, Isoleucyl-Alanine, 2-(2,4-dihydroxyphenyl)-5,7-dihydroxy-6-(3-methylbut-2-en-1-yl)-3,4-dihydro-2H-1-benzopyran-4-one, N-(2-Phenylethyl)-acetamide, L-cis-Cyclo(aspartylphenylalanyl, leucyl-Alanine, sulfolithocholylglycine, curcumadiol, caryoptosidic acid, and ascorbyl stearate. The 30 metabolites above mentioned could be divided into six categories, including lipids and lipid-like molecules (12), organic acids and derivatives (10), organic oxygen compounds (3), nucleosides, nucleotides, analogs (2), organoheterocyclic compounds (1), phenylpropanoids and polyketides (1), and organic nitrogen compounds (1). Particularly, 8 of the 30 compounds with high content in BH of the trial group were dipeptides. However, 76 nVFC decreased significantly in the sample of SF3 (VIP > 1.0, *p* < 0.05, FC < 0.5), acetylcholine and 1-Methylhypoxanthine reduced by more than 32 times (Figure 5).

### Correlation between fungi and nVFCs in CK3 and SF3

**Figure 6:**
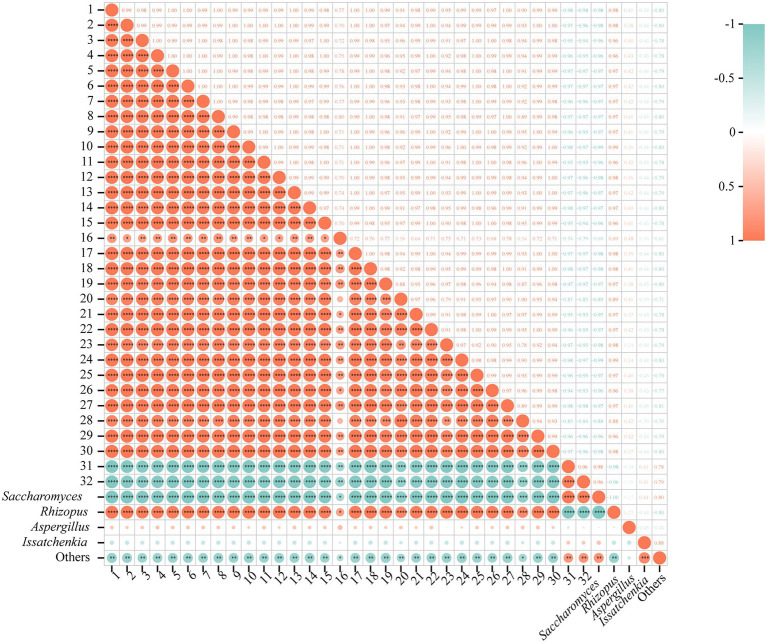
Correlation between fungi and nVFCs. 1 ~ 32, same as Figure 5. ^*^p < 0.05, ^**^p < 0.01, ^***^p < 0.001, ^****^p < 0.0001.

The analysis of correlation between fungi and nVFCs was exhibited at the genus level (Figure 6). It revealed that *Saccharomyces* showed significant negative correlation with the contents of nVFCs numbered as 1 to 30, such as prolyl-valine, lysoPE (0:0/22:0), enkephalin, (D-Ala)2-Leu, and agavoside A (*r* > 0.80, *p* < 0.01), while the contrary trend was just discovered for acetylcholine and 1-Methylhypoxanthine (*r* > 0.80, *p* < 0.01). Interestingly, relative abundance of *Rhizopus* was significant positive correlation with the contents of nVFCs numbered as 1 to 30 (*r* > 0.80, *p* < 0.01), but a significant negative correlation with acetylcholine and 1-Methylhypoxanthine (*r* > 0.80, *p* < 0.01). In a word, the correlation rules with contents of 32 metabolites were just opposite for *Saccharomyces* and *Rhizopus*. The relative abundance of *Aspergillus* and *Issatchenkia* was not correlated with the contents of these 32 nVFCs (*p* > 0.05). Whereas, the coincident pattern of change was true for the correlation between the relative richness of other fungus and amounts of 32 nVFC (*r* > 0.70, *p* < 0.05). In conclusion, the relative abundance of *Saccharomyces* and *Rhizopus* had a significant effect on the content of differential metabolites in BH.

## Discussion

It was reported that the final flavor quality of *Huangjiu* was determined by the microbial species, relative abundances, and their interactions in the starter. In the proceeding of cellular metabolism, an array of enzymes are generated by *Rhizopus* and *Saccharomyces*, which catalyze starch into various small molecule compounds, such as available sugars, resulting the reinforcement of product flavor (Yang et al., 2017). *Rhizopus* and *Saccharomyces* were common microorganisms used to make *Huangjiu*. In traditional fermentation process, they were added simultaneously to the fermentation feedstock (Cai et al., 2018; Chen et al., 2021). In this case, however, the saccharification was often incomplete (Chen and Xu, 2013). For the sequential inoculation, the raw material was firstly partially saccharified by *Rhizopus* to form a certain amount of sugar, which was provided to *Saccharomyces* for their growth and metabolism.

This study employed two different inoculation methods (simultaneous inoculation and sequential inoculation) to explore the differences in fungal communities during BH fermentation. The results showed that different inoculation methods significantly affected the microbial diversity of BH. *Saccharomyces* and *Rhizopus* were the main dominant fungal genera in CK and SF, which was consistent with previous reports (Jiang et al., 2020; Zhao et al., 2020). For the stage of CK1 to CK2, the fungal richness of sample showed a trend of rising and then falling, while there was no significant difference in the fungal diversity. A plausible possibility was that the sugar derived from starch hydrolysis was immediately utilized by the *Saccharomyces*. However, the *Saccharomyces* was rapidly proliferated in the stage of CK2 to CK3, which led the accumulation of alcohol and further inhibited the growth of other fungi.

In the sequential inoculation group (SF), the fungal community richness was significantly decreased, but completely opposite for the fungal diversity. A large amount of starch was hydrolyzed by the *Rhizopus* to generate high concentration of glucose in the SF1 to SF2 stage. Subsequently, the growth of *Saccharomyces* was inhibited because of the high osmotic pressure in the fermentation stage of SF2-SF3, while the *Rhizopus* grew moderately. Moreover, the relative richness of *Rhizopus* was contrary to that of *Saccharomyces* in CK3 and SF3 (Figure 2B), suggesting a competitive relationship between them during fermentation. In addition, very few of *Aspergillus* and *Issatchenkia* were detected in the sample of BH, which could be from air due to this work was carried out in an open environment.

Correlation analysis demonstrated that *Saccharomyces* and *Rhizopus* were significantly correlated with the content of organic acid, and this point clearly supported previous studies (Peleg et al., 1988; Pines et al., 1996). Compared with CK3, the amount of lipids and lipid-like molecules (12) was significantly decreased, but quite reverse trend was found for that of organic acids and derivatives (10) as well as organic oxygen compounds (3) in SF3. It was speculated that *Saccharomyces* and *Rhizopus* could grow rapidly and maintain a high level of diversity and activity because of abundant nutrients, thereby increasing the flavor compounds synthesized by microbe metabolism after *Saccharomyces* be inoculated in the sequential fermentation. However, high concentration of alcohols biosynthesized by *Saccharomyces* was detected in the fermentation liquor of CK3, wherein fewer flavor substances were produced and the diversity of fungus was weakened due to the other microorganisms be suppressed by yeast in this case.

A variety of metabolites, such as sugar, organic acids, amino acids, and peptides, were responsible for the unique taste of *Huangjiu* (Yu et al., 2015). Among which, sugars (the main source of sweetness), such as caramel, arabinose, etc., mainly derived from the utilization and transformation of carbohydrates and proteins by microorganisms (Chen et al., 2021). It was found that the special BH adding mulberry juice could not only provide available sugars required for the growth and metabolism of microorganisms, but also the precursor and active compounds to generate the pleasant taste in our previous research. Interestingly, as a dipeptide, Asp-Phe was significantly increased in the sample of SF3. Published data proved that it was an artificial sweetener (Grobelny and Galardy, 1985). And thus, the sweetness of SF3 sample was more intense. Organic acids and some amino acids, including proline, glutamic acid, isoleucine, threonine, and lysine, were the main source of sour of *Huangjiu*. The former was mainly a result of the metabolism of *Saccharomyces* and *Lactobacillus* (Chen et al., 2021). But for the latter, they mainly derived from carbohydrate, protein conversion, and microbial fermentation in *Huangjiu* (Yu et al., 2015). In general, appropriate sour compounds could enhance the sweet and refreshing flavor of BH (Chen et al., 2021). The types and contents of organic acids and their derivatives were significantly increased in SF3, so the acidity of SF3 was more prominent than that of CK3. Several reports demonstrated that the amargosa were mainly due to a set of compounds produced during the fermentation process, such as polypeptides, arginine, valine, and their derivatives. Among which, the amino acid sequence of a key bitter peptide was found and named as Leu-ProThr-Leu (Xie, 2021). However, the primary bitter amino acids (arginine and valine) and key bitter peptides were not detected in all samples of the control and experimental groups. In addition, esters and derivatives in the CK3 group were significantly higher than those in the SF3 group, of which some esters contributed to the flavor of BH. It was worth noting that Gamma-Glutamylphenylalanine was significantly increased in SF3. What made people excited was that it was a “kokumi” flavor substance according to reports (Sgarbi et al., 2013; Kuroda and Miyamura, 2015). Therefore, the sample of SF3 group was more mellow and there was a clear difference in flavor between the two groups.

Mulberry juice was rich in sugars and flavonoids (anthocyanins). It was well known that sugars could be served as fermentation substrates for *Saccharomyces* to produce ethanol. As an important antioxidant, flavonoids may play an important role in maintaining the color of BH. Overall, this work clarified the complex relationship between microbes and non-volatile favor components formed during the fermentation of BH. Thus, the final taste of BH may be a consequence of synergistic effects of microbial taxa. In future studies, much work remains in order to elaborate the function of *Rhizopus* in BH fermentation by transcriptome and other food omics analysis methods.

## Conclusion

In this study, we compared the fungal communities and nVFCs during BH fermentation. It was found that the content of main taste substances (organic acids and derivatives) in the BH of sequential inoculation fermentation increased significantly (*p* < 0.05) compared with traditional fermentation, while the content of lipids and lipids-like molecules decreased significantly (*p* < 0.05). The metabolic profiling analysis of fungal communities found that lipids and organic acids were significantly (*p* < 0.05) negatively correlated with *Saccharomyces*, and significantly (*p* < 0.05) positively correlated with *Rhizopus*. In a word, compared with simultaneous inoculation, BH fermented by sequential inoculation, the taste was stronger, sweeter, mellow and softer. This study can provide practical and effective experimental basis for improving the quality of BH and optimizing its fermentation process.

## Data availability statement

The original contributions presented in the study are included in the article/Supplementary Material. Further inquiries can be directed to the corresponding authors.

## Author contributions

KL and WY conceived this study and participated in its design and coordination. PL and RS designed the experiments and drafted the manuscript. QW, HY, and BX performed the experiments. WD and ZL analyzed the data. SC and WY funded and supervised the experiments. All authors contributed to the article and approved the submitted version.

## Funding

This research was funded by the National Key R&D Program of China, grant number: 2021YFD1100403; Major Science and Technology Projects in Yunnan Province of China, grant number: 202102AE090010, and supported by China Agriculture Research System of MOF and MARA; Scientific Research Project of Yibin Vocational and Technical College, grant number: ZRKY21ZD-04; and Science and Technology Innovation Team Project of Yibin Vocational and Technical College, grant number: ybzy21cxtd-03.

## Conflict of interest

BX is employed by Luzhou Greenland Wine Co., Ltd.

The remaining authors declare that the research was conducted in the absence of any commercial or financial relationships that could be construed as a potential conflict of interest.

## Publisher’s note

All claims expressed in this article are solely those of the authors and do not necessarily represent those of their affiliated organizations, or those of the publisher, the editors and the reviewers. Any product that may be evaluated in this article, or claim that may be made by its manufacturer, is not guaranteed or endorsed by the publisher.

## Footnotes

^1^ https://github.com/OpenGene/fastp

^2^ http://drive5.com/uparse/

^3^ http://sourceforge.net/projects/rdp-classifier/

^4^ http://unite.ut.ee/

^5^ https://www.metaboanalyst.ca/

^6^ www.ncbi.nlm.nih.gov/bioproject

## Supplementary materials

The Supplementary Materials for this article can be found online at: https://www.frontiersin.org/articles/10.3389/fmicb.2022.955825/full#supplementary-material
