# Supplementary material for: Comparison of fungal communities and nonvolatile flavor components in black Huangjiu formed using different inoculation fermentation methods
Source: Front Microbiol. 2022 Jul 22;13:955825. doi: 10.3389/fmicb.2022.955825 (PMC9354453; doi:10.3389/fmicb.2022.955825)
Supplement: Supplementary file 1 [file Table_1.DOCX]

Table S1. Metabolite Superclass relative abundance in CK3 and SF3 (%)

| HMDB Superclass | Number | CK3 | SF3 |
| --- | --- | --- | --- |
| Lipids and lipid-like molecules | 197 | 43.23±0.08a | 42.69±0.10b |
| Organic acids and derivatives | 83 | 16.49±0.05b | 17.08±0.04a |
| Organic oxygen compounds | 60 | 13.43±0.06b | 13.67±0.08a |
| Organoheterocyclic compounds | 44 | 8.78±0.04a | 8.44±0.03b |
| Phenylpropanoids and polyketides | 40 | 8.29±0.05a | 8.14±0.07b |
| Benzenoids | 29 | 5.93±0.03a | 5.88±0.01b |
| Organic nitrogen compounds | 8 | 1.99±0.02a | 1.86±0.01b |
| Nucleosides, nucleotides, and analogues | 8 | 1.40±0.12b | 1.79±0.01a |
| Homogeneous non-metal compounds | 1 | 0.25±0.00a | 0.25±0.00a |
| Alkaloids and derivatives | 1 | 0.21±0.00a | 0.19±0.00b |

Different lowercase letters in the same row indicate extremely significant differences (*P* < 0.01).

Table S2. Candidate ruminal metabolites that differed between the control (CK3) and the treatment (SF3).

| Metabolite | VIP_pred_OPLS-DA | VIP_PLS-DA | FC(SF3/CK3) | FDR |
| --- | --- | --- | --- | --- |
| 9,12,13-TriHOME | 1.007457 | 1.002469 | 0.918568 | 7.20E-07 |
| Deoxyribose | 1.208197 | 1.206611 | 1.146565 | 1.15E-12 |
| 3-Hydroxyadipic acid | 1.059267 | 1.059482 | 1.089739 | 5.29E-11 |
| (R)-3-((R)-3-Hydroxybutanoyloxy)butanoate | 1.658696 | 1.65435 | 1.387676 | 9.86E-12 |
| 13,14-Dihydro PGF-1a | 1.492109 | 1.492942 | 1.252671 | 1.84E-06 |
| (Z)-15-Oxo-11-eicosenoic acid | 1.20717 | 1.20528 | 1.132233 | 3.00E-06 |
| 9-hydroxy-5Z-nonenoic acid | 1.014544 | 1.009087 | 0.911532 | 1.70E-07 |
| Xi-7-Hydroxyhexadecanedioic acid | 1.47683 | 1.474428 | 1.286108 | 5.51E-09 |
| Blumenol C O-[rhamnosyl-(1->6)-glucoside] | 1.316712 | 1.310392 | 1.201597 | 4.04E-07 |
| N2-(D-1-Carboxyethyl)-L-lysine | 1.433641 | 1.42928 | 1.261538 | 7.87E-11 |
| D-Glucaro-1,4-lactone | 1.216626 | 1.212747 | 1.150916 | 3.28E-06 |
| Starch acetate | 1.007885 | 1.008991 | 1.095673 | 6.58E-10 |
| Occidentoside | 1.010472 | 1.009398 | 1.100448 | 2.73E-07 |
| Guanosine | 1.218466 | 1.207717 | 1.218105 | 0.001273 |
| PS(MonoMe(11,5)/MonoMe(11,3)) | 1.952168 | 1.943878 | 1.55405 | 1.89E-09 |
| Agavoside A | 2.226687 | 2.215202 | 1.865173 | 3.58E-08 |
| 6-{[(16S)-5,7-dihydroxy-8,8,10,16-tetramethyl-3-[1-(2-methyl-1,3-thiazol-4-yl)prop-1-en-2-yl]-12-methylidene-9-oxo-17-oxa-4-azabicyclo[14.1.0]heptadec-4-en-11-yl]oxy}-3,4,5-trihydroxyoxane-2-carboxylic acid | 2.114325 | 2.10763 | 1.728468 | 4.48E-11 |
| (E)-2-Hexenyl (E)-7,9-decadienoate | 1.18676 | 1.187766 | 1.201285 | 1.56E-06 |
| 17-Hydroxy-E4-neuroprostane | 1.01197 | 1.006835 | 0.894726 | 1.22E-05 |
| Phytocassane C | 1.190964 | 1.186128 | 0.871115 | 2.03E-05 |
| 9,10-epoxy-12-octadecenoic acid | 1.063204 | 1.058288 | 0.904014 | 2.99E-06 |
| LysoPA(0:0/18:1(9Z)) | 1.55739 | 1.553351 | 1.291871 | 6.02E-06 |
| Alanyl-Proline | 1.526948 | 1.52278 | 1.291589 | 9.23E-12 |
| Hypoxanthine | 2.252921 | 2.246555 | 0.59183 | 1.59E-09 |
| D-Mannose 6-phosphate | 1.134891 | 1.134679 | 1.148915 | 1.42E-09 |
| N-Acetylmannosamine | 1.081132 | 1.080002 | 1.11736 | 2.82E-10 |
| 5-Methylcytosine | 1.326548 | 1.321117 | 0.813582 | 8.08E-10 |
| ETHYL-p-COUMARATE | 1.2164 | 1.215534 | 1.20767 | 2.86E-09 |
| Natamycin | 2.22356 | 2.215772 | 1.893389 | 2.06E-09 |
| Tyrosyl-Valine | 1.199074 | 1.193889 | 1.180742 | 1.27E-07 |
| Physapruin B | 1.933333 | 1.927356 | 1.526269 | 1.06E-10 |
| Enkephaline, (D-Ala)2-Leu | 2.251568 | 2.242684 | 1.999583 | 2.20E-09 |
| 12-Oxo-2,3-dinor-10,15-phytodienoic acid | 1.65273 | 1.64927 | 1.457663 | 5.94E-08 |
| 7,10-Hexadecadiynoic acid | 1.346089 | 1.34442 | 1.240033 | 3.93E-08 |
| Isoprothiolane | 1.422077 | 1.418207 | 0.791826 | 1.29E-06 |
| PGE2 1,15-lactone | 1.102542 | 1.095708 | 0.901774 | 1.50E-06 |
| 12,13-DHOME | 1.123377 | 1.115761 | 0.905038 | 4.76E-07 |
| Austroinulin | 1.146724 | 1.145461 | 1.132607 | 2.59E-06 |
| 13(S)-HODE methyl ester | 1.137433 | 1.14245 | 1.15132 | 4.39E-05 |
| Methyl 9,10-epoxy-12,15-octadecadienoate | 1.397327 | 1.399023 | 1.240967 | 6.91E-06 |
| Phosphocholine | 1.240714 | 1.238579 | 1.148726 | 7.06E-13 |
| Prenyl glucoside | 1.081655 | 1.07841 | 1.112736 | 6.41E-09 |
| Isonicotineamide | 1.554968 | 1.551915 | 1.331021 | 7.84E-10 |
| Stachyose | 1.198498 | 1.198441 | 1.131294 | 1.08E-07 |
| Isoleucyl-Valine | 1.509773 | 1.505391 | 1.284052 | 1.27E-11 |
| 3,5-dihydroxybenzoic acid | 1.162998 | 1.161558 | 1.185669 | 4.04E-11 |
| 4-{hydroxy[(3,4,5,6-tetrahydroxyoxan-2-yl)methoxy]methylidene}cyclohexa-2,5-dien-1-one | 1.269214 | 1.266047 | 1.214032 | 1.24E-06 |
| Phenylalanyl-Valine | 1.379057 | 1.377179 | 1.320098 | 5.06E-08 |
| Phenylethylamine | 1.576218 | 1.5734 | 1.373883 | 1.35E-12 |
| LysoPE(0:0/22:0) | 2.330044 | 2.323484 | 2.162439 | 2.76E-07 |
| Dihydroceramide C2 | 1.003268 | 1.004617 | 1.121119 | 2.70E-05 |
| 11-peroxy-5Z,8Z,12E,14Z-eicosatetraenoate | 1.156268 | 1.150458 | 0.888581 | 6.08E-07 |
| 4-Androstenediol | 1.283505 | 1.281174 | 1.159895 | 3.76E-07 |
| 7a-Hydroxy-5b-cholanic acid | 1.890018 | 1.883466 | 1.593675 | 8.37E-07 |
| 3,4-Dimethyl-5-propyl-2-furantridecanoic acid | 1.578981 | 1.579032 | 1.365093 | 4.28E-06 |
| 13(S)-HOTrE | 1.07902 | 1.078973 | 1.111673 | 5.21E-06 |
| Gamma-Linolenic Acid ethyl ester | 1.214943 | 1.215474 | 1.142521 | 3.51E-06 |
| Methyl linolenate | 1.01913 | 1.037058 | 1.14991 | 0.001119 |
| 9-HETE | 1.276416 | 1.2709 | 0.839878 | 1.03E-06 |
| Stearidonic Acid ethyl ester | 1.034289 | 1.045159 | 1.127746 | 0.000165 |
| 4-Methyl-1-phenyl-2-pentanol | 1.164864 | 1.157046 | 0.863257 | 1.51E-05 |
| 3-ketosphinganine | 1.31812 | 1.318435 | 1.204087 | 1.85E-08 |
| Arginyl-Glutamine | 1.267229 | 1.269913 | 1.20005 | 1.01E-05 |
| 11-deoxy-PGE1 | 1.28812 | 1.290435 | 1.203833 | 4.28E-06 |
| (S)-9-Hydroxy-10-undecenoic acid | 1.140841 | 1.147007 | 1.16038 | 3.32E-05 |
| (1R,2R)-3-oxo-2-pentyl-cyclopentanehexanoic acid | 1.394132 | 1.393153 | 1.292842 | 1.36E-06 |
| (+/-)-Octanoylcarnitine | 1.875587 | 1.872176 | 1.504095 | 4.82E-11 |
| N-(2-Phenylethyl)-acetamide | 1.860858 | 1.857492 | 1.572581 | 4.51E-12 |
| L-cis-Cyclo(aspartylphenylalanyl) | 1.835618 | 1.831038 | 1.532976 | 1.84E-10 |
| Leucyl-Alanine | 1.818912 | 1.814893 | 1.801125 | 8.30E-10 |
| Isoleucyl-Serine | 1.439071 | 1.434548 | 1.305175 | 1.15E-09 |
| 4-Oxoretinol | 2.479355 | 2.469873 | 3.306604 | 6.54E-08 |
| Glycylleucine | 1.530885 | 1.525785 | 1.35939 | 7.37E-10 |
| Isoleucyl-Alanine | 1.870061 | 1.865264 | 1.675952 | 8.48E-11 |
| Ethyl vanillin | 1.159408 | 1.154801 | 0.848427 | 1.04E-08 |
| (+/-)-Hexanoylcarnitine | 1.991847 | 1.98705 | 1.691994 | 2.84E-13 |
| Isoleucyl-Leucine | 1.973606 | 1.967351 | 1.655826 | 3.29E-11 |
| Threoninyl-Valine | 1.445494 | 1.440899 | 1.318079 | 5.42E-10 |
| Isoleucyl-Isoleucine | 1.931308 | 1.925536 | 1.531799 | 7.01E-12 |
| Tyramine | 1.482504 | 1.47752 | 1.29203 | 1.44E-10 |
| 2-Oxo-1,2-dihydroquinoline-4-carboxylate | 1.041505 | 1.038448 | 1.150437 | 1.39E-08 |
| N-Acetyl desmethyl frovatriptan | 2.625642 | 2.608877 | 3.377903 | 6.20E-06 |
| Asp-Phe | 2.039912 | 2.033003 | 1.798453 | 2.30E-10 |
| L-Tyrosine | 1.398712 | 1.394561 | 1.288432 | 8.57E-10 |
| N-Ethyl trans-2-cis-6-nonadienamide | 1.65155 | 1.645882 | 1.392912 | 2.16E-10 |
| 2'-O-Methyladenosine | 1.332011 | 1.327231 | 1.200604 | 1.64E-06 |
| 1-Methylhypoxanthine | 1.048807 | 1.042162 | 0.855498 | 4.62E-07 |
| Adenosine 3'-monophosphate | 2.011174 | 1.997501 | 1.559006 | 5.91E-06 |
| D[-Arg-2]KYOTORPHAN | 1.5647 | 1.559607 | 1.325084 | 2.53E-10 |
| Nalpha-Acetyl-L-arginine | 1.287283 | 1.283312 | 1.202122 | 1.91E-10 |
| Acetylcholine | 1.811493 | 1.80782 | 0.725431 | 3.04E-12 |
| Niacinamide | 1.320985 | 1.319206 | 1.193496 | 9.39E-10 |
| (S)C(S)S-S-Methylcysteine sulfoxide | 1.157711 | 1.155874 | 0.850142 | 6.40E-11 |
| 2-(2,4-dihydroxyphenyl)-5,7-dihydroxy-6-(3-methylbut-2-en-1-yl)-3,4-dihydro-2H-1-benzopyran-4-one | 1.869738 | 1.868339 | 1.516309 | 6.44E-09 |
| 2-Phenylaminoadenosine | 2.654355 | 2.655832 | 3.311254 | 4.31E-05 |
| 3-Acetyldihydro-2(3H)-furanone | 1.507083 | 1.504555 | 1.255182 | 2.54E-11 |
| 5-Methyl-2-furaldehyde | 1.461859 | 1.46001 | 1.252243 | 1.45E-09 |
| 2-Pyrrolidinone | 1.470163 | 1.46689 | 0.770617 | 6.22E-11 |
| Glycerylphosphorylethanolamine | 1.283972 | 1.281059 | 1.190056 | 4.86E-13 |
| Isonicotinic acid | 1.062849 | 1.059713 | 0.90015 | 3.85E-10 |
| N-Acetylneuraminic acid | 1.093787 | 1.094877 | 1.112052 | 2.48E-09 |
| Zeranol | 1.303689 | 1.302677 | 1.241288 | 1.04E-07 |
| Galactosylglycerol | 1.659665 | 1.652985 | 1.331172 | 3.43E-09 |
| Guanidylic acid (guanosine monophosphate) | 1.747621 | 1.739303 | 1.302686 | 2.71E-08 |
| 3-Hydroxydodecanedioic acid | 1.749607 | 1.742811 | 0.721195 | 1.67E-12 |
| 2,4-Dimethylpimelic acid | 1.26886 | 1.262597 | 0.873378 | 9.43E-08 |
| 9,10,13-TriHOME | 1.027029 | 1.02185 | 0.931464 | 6.81E-07 |
| (9S,10S)-9,10-dihydroxyoctadecanoate | 1.27119 | 1.261907 | 0.87616 | 2.51E-06 |
| Gamma-Glutamylphenylalanine | 2.250915 | 2.241902 | 1.883368 | 2.40E-11 |
| Alpha-D-Galacturonic acid | 1.655269 | 1.649535 | 1.254876 | 3.88E-12 |
| Uridine diphosphate-N-acetylglucosamine | 2.091471 | 2.0812 | 1.689607 | 1.20E-05 |
| N-Acetyl-L-glutamic acid | 1.157893 | 1.153103 | 1.140984 | 1.55E-10 |
| 8-[(Aminomethyl)sulfanyl]-6-sulfanyloctanoic acid | 1.170407 | 1.16153 | 1.16514 | 6.83E-05 |
| Adipic acid | 1.326554 | 1.321841 | 1.204701 | 5.27E-10 |
| [2-hydroxy-5-(3,5,7-trihydroxy-4-oxo-3,4-dihydro-2H-1-benzopyran-2-yl)phenyl]oxidanesulfonic acid | 1.603835 | 1.597525 | 0.752368 | 3.75E-09 |
| 2-Ethyl-2-Hydroxybutyric acid | 1.305058 | 1.300373 | 1.197655 | 5.35E-11 |
| 2-Isopropylmalic acid | 1.24699 | 1.242404 | 1.129557 | 4.86E-11 |
| 6-{[(16S)-5,7-dihydroxy-8,8,12,16-tetramethyl-3-[1-(2-methyl-1,3-thiazol-4-yl)prop-1-en-2-yl]-10-methylidene-9-oxo-17-oxa-4-azabicyclo[14.1.0]heptadec-4-en-11-yl]oxy}-3,4,5-trihydroxyoxane-2-carboxylic acid | 2.282736 | 2.272217 | 1.840651 | 2.96E-09 |
| 6-{[3-(2,4-dihydroxy-5-methoxyphenyl)propanoyl]oxy}-3,4,5-trihydroxyoxane-2-carboxylic acid | 1.131578 | 1.125508 | 1.155358 | 1.69E-05 |
| Suberic acid | 1.342969 | 1.336956 | 0.821387 | 5.13E-08 |
| 2-Hydroxydecanedioic acid | 1.05854 | 1.052889 | 0.893404 | 4.44E-08 |
| Polyethylene, oxidized | 1.11918 | 1.113012 | 0.889608 | 8.65E-07 |
| (+/-)-3-Hydroxynonanoic acid | 1.800362 | 1.790844 | 0.684476 | 1.73E-10 |
| 9,10-DiHODE | 1.282751 | 1.274229 | 0.872883 | 5.13E-07 |
| 15-Keto-13,14-dihydroprostaglandin A2 | 1.316806 | 1.307919 | 0.836183 | 1.51E-06 |
| Sterebin E | 1.263895 | 1.254608 | 0.851899 | 4.02E-06 |
| 13(S)-HODE | 1.356709 | 1.351858 | 0.872741 | 1.38E-05 |
| LysoPA(0:0/18:2(9Z,12Z)) | 1.374321 | 1.375345 | 1.176674 | 4.43E-05 |
| 3,4,5-trihydroxy-6-{[8-methoxy-6-(3-methylbut-2-en-1-yl)-2-oxo-2H-chromen-7-yl]oxy}oxane-2-carboxylic acid | 1.591427 | 1.584176 | 1.31297 | 8.54E-09 |
| D-Glyceraldehyde 3-phosphate | 1.006523 | 1.002203 | 1.084684 | 2.01E-06 |
| 3-Hydroxyadipic acid 3,6-lactone | 1.240047 | 1.239694 | 1.145333 | 1.77E-10 |
| Glucose 1-phosphate | 1.2547 | 1.249296 | 1.117421 | 2.52E-09 |
| 2-Hydroxyadenine | 1.080642 | 1.079158 | 0.879248 | 9.04E-09 |
| 5'-CMP | 1.296246 | 1.286615 | 1.182785 | 1.89E-05 |
| Citric acid | 1.235524 | 1.228613 | 1.125228 | 1.29E-05 |
| L-4-Hydroxyglutamate semialdehyde | 1.509455 | 1.50759 | 0.802366 | 3.88E-12 |
| Xanthine | 1.28653 | 1.280799 | 0.856031 | 7.70E-11 |
| Uridine monophosphate (UMP) | 1.245629 | 1.236548 | 1.149332 | 3.52E-06 |
| Molybdopterin precursor Z | 1.115225 | 1.110776 | 0.851022 | 0.000404 |
| 4-HYDROXY-6-METHYLPYRAN-2-ONE | 1.151545 | 1.146547 | 1.177278 | 1.14E-08 |
| N-Acetyl-9-O-acetylneuraminic acid | 1.100334 | 1.095705 | 1.135927 | 6.45E-06 |
| LysoPE(0:0/15:0) | 2.165903 | 2.158116 | 1.921271 | 1.21E-09 |
| Valyl-Isoleucine | 1.433087 | 1.42661 | 1.308943 | 6.25E-10 |
| N-Acetyl-5-aminosalicylic acid | 2.090822 | 2.086105 | 2.239549 | 1.06E-09 |
| Caryoptosidic acid | 1.894926 | 1.885194 | 1.608541 | 3.07E-08 |
| Prolyl-Valine | 2.439295 | 2.431597 | 2.062683 | 2.93E-09 |
| Dihydrozeatin-7-N-dihydrozeatin | 2.300755 | 2.293741 | 1.946128 | 6.02E-13 |
| Glutamylisoleucine | 1.082522 | 1.075142 | 1.138124 | 4.51E-06 |
| 3,4,5-trihydroxy-6-[(2-phenylpropanoyl)oxy]oxane-2-carboxylic acid | 1.276103 | 1.269573 | 1.174402 | 2.00E-06 |
| Ganoderic acid Mk | 2.367556 | 2.358833 | 2.070657 | 4.60E-12 |
| 1-(4-Methoxyphenyl)-1-penten-3-one | 1.486024 | 1.479347 | 1.274832 | 1.31E-10 |
| Kiwiionoside | 2.220284 | 2.212129 | 1.999045 | 1.86E-12 |
| Physagulin D | 2.116131 | 2.106459 | 1.688471 | 6.03E-10 |
| Deoxyloganic acid | 3.128261 | 3.118379 | 0.000253 | 5.97E-12 |
| Corchorifatty acid D | 1.359192 | 1.350223 | 0.819324 | 6.33E-08 |
| Prostaglandin E2 | 1.01302 | 1.007779 | 0.919223 | 7.26E-07 |
| Ipomeatetrahydrofuran | 1.647777 | 1.643614 | 1.337861 | 2.37E-08 |
| Avocadene | 1.321369 | 1.323053 | 1.188357 | 6.71E-06 |
| Cis-9,10-Epoxystearic acid | 1.544551 | 1.53507 | 0.828653 | 1.05E-07 |
| 1-Stearoylglycerophosphoserine | 1.014752 | 1.036626 | 1.134317 | 0.001343 |
| PS(18:1(9Z)/0:0) | 1.506833 | 1.511041 | 1.34926 | 3.61E-05 |
| (R)-6'-O-(4-Geranyloxy-2-hydroxycinnamoyl)-marmin | 1.625776 | 1.630334 | 1.387531 | 9.25E-05 |
| DG(15:0/18:3(6Z,9Z,12Z)/0:0) | 1.754487 | 1.746022 | 0.684444 | 7.35E-05 |
| Diepomuricanin A | 1.244302 | 1.235642 | 0.855121 | 1.84E-05 |
| 1-Oleoyl Lysophosphatidic Acid (sodium salt) | 1.514311 | 1.508996 | 1.227969 | 1.09E-05 |
| 1-(11Z-eicosenoyl)-glycero-3-phosphate | 1.066606 | 1.085937 | 1.133302 | 0.000705 |
| 1-Oleoylglycerophosphoinositol | 1.395231 | 1.405901 | 1.204113 | 0.000232 |
| Methyl linoleate | 2.013655 | 1.998611 | 0.527574 | 7.69E-06 |
| 13-OxoODE | 1.305174 | 1.299367 | 0.856607 | 1.89E-05 |
| PE(18:3/0:0) | 1.074647 | 1.115932 | 1.1575 | 0.003725 |
| 9(S)-HOTrE | 1.269979 | 1.264407 | 0.861138 | 1.41E-05 |
| 12,20-DiHETE | 1.19317 | 1.18448 | 0.881503 | 4.47E-07 |
| 9,10-DiHOME | 1.235481 | 1.2265 | 0.895615 | 2.26E-07 |
| (+)-15,16-Dihydroxyoctadecanoic acid | 1.715115 | 1.705976 | 0.750891 | 2.02E-08 |
| 9-Pentadecenoic acid | 1.550696 | 1.547746 | 1.304715 | 3.46E-07 |
| 2,10-Bisaboladiene-1,4-diol | 1.965568 | 1.958642 | 1.516703 | 7.07E-09 |
| Ethyl 3-hydroxytridecanoate | 2.388133 | 2.377467 | 2.358647 | 4.40E-08 |
| Curcumadiol | 1.874161 | 1.867457 | 1.419238 | 1.73E-08 |
| 9(S)-HpOTrE | 1.259665 | 1.251049 | 0.856542 | 1.37E-07 |
| 5-hydroperoxy-15-HETE | 1.182244 | 1.174569 | 0.879984 | 6.05E-08 |
| 11-Hydroxy-9-tridecenoic acid | 1.279191 | 1.275282 | 1.200458 | 4.38E-09 |
| P-Salicylic acid | 1.036562 | 1.03035 | 0.90299 | 4.46E-07 |
| Ascorbyl stearate | 1.874713 | 1.864717 | 1.567069 | 2.55E-08 |
| PG(i-12:0/18:2(9Z,11Z)) | 2.299403 | 2.291305 | 1.976491 | 5.69E-13 |
| Piperolein B | 1.76896 | 1.762443 | 1.459945 | 7.73E-11 |
| Fumitremorgin B | 2.280957 | 2.272154 | 2.067182 | 5.54E-12 |
| Succinylacetone | 1.335965 | 1.331978 | 1.18197 | 1.97E-10 |
| 3-Isopropylmalate | 1.584915 | 1.577099 | 1.321144 | 4.34E-07 |
| Osmundalactone | 1.79041 | 1.782366 | 0.731529 | 2.85E-11 |
| Cinncassiol D1 glucoside | 2.25345 | 2.245345 | 2.038573 | 1.34E-12 |
| (3,4,5,6-tetrahydroxyoxan-2-yl)methyl 4-hydroxybenzoate | 1.262207 | 1.258287 | 1.186569 | 9.46E-08 |
| Isoleucyl-Tyrosine | 2.016599 | 2.008741 | 1.687475 | 1.50E-10 |
| Serylisoleucine | 1.54444 | 1.536017 | 1.355867 | 4.53E-09 |
| 3'-Hydroxyhexobarbital | 1.690098 | 1.681555 | 1.557904 | 6.19E-10 |
| N-Acetyl-L-alanine | 1.313283 | 1.309224 | 1.227118 | 3.28E-13 |
| Ribose 1-phosphate | 1.311087 | 1.302283 | 1.177186 | 4.14E-06 |
| Adenine | 1.571547 | 1.571114 | 0.756176 | 4.99E-09 |
| Oxoglutaric acid | 1.666594 | 1.664077 | 1.349941 | 2.01E-08 |
| 6-ethoxy-3,4,5-trihydroxyoxane-2-carboxylic acid | 1.323934 | 1.315831 | 1.183634 | 9.26E-07 |
| 6-[4-(2-carboxyethyl)-5-hydroxy-2-methoxyphenoxy]-3,4,5-trihydroxyoxane-2-carboxylic acid | 1.871918 | 1.87499 | 0.686533 | 3.51E-09 |
| Digalacturonate | 2.016004 | 2.007768 | 1.439828 | 5.89E-09 |
| 1-(sn-Glycero-3-phospho)-1D-myo-inositol | 1.085296 | 1.079853 | 1.091973 | 1.46E-08 |
| 1D-Myo-inositol 1,4-bisphosphate | 1.128081 | 1.122072 | 1.150651 | 1.51E-06 |
| L-Arginine | 1.102255 | 1.104219 | 0.87589 | 4.63E-08 |
| Aconitic acid | 1.152758 | 1.147151 | 1.138004 | 2.31E-06 |
| Caffeic Acid | 1.000608 | 0.999155 | 1.109627 | 3.25E-07 |
| Dihydrobiopterin | 1.545327 | 1.5482 | 0.754839 | 7.25E-09 |
| Dulcitol | 1.835178 | 1.829305 | 1.302121 | 8.88E-09 |
| EPIGALLOCATECHIN | 1.549328 | 1.542948 | 0.789742 | 1.86E-07 |
| Malic acid | 1.109192 | 1.102851 | 1.10153 | 2.03E-07 |
| Maltose | 1.03965 | 1.039308 | 1.071275 | 1.28E-08 |
| Ornithine | 1.112908 | 1.108663 | 0.859092 | 2.07E-07 |
| Sulfolithocholylglycine | 1.909791 | 1.901978 | 1.544123 | 8.14E-09 |


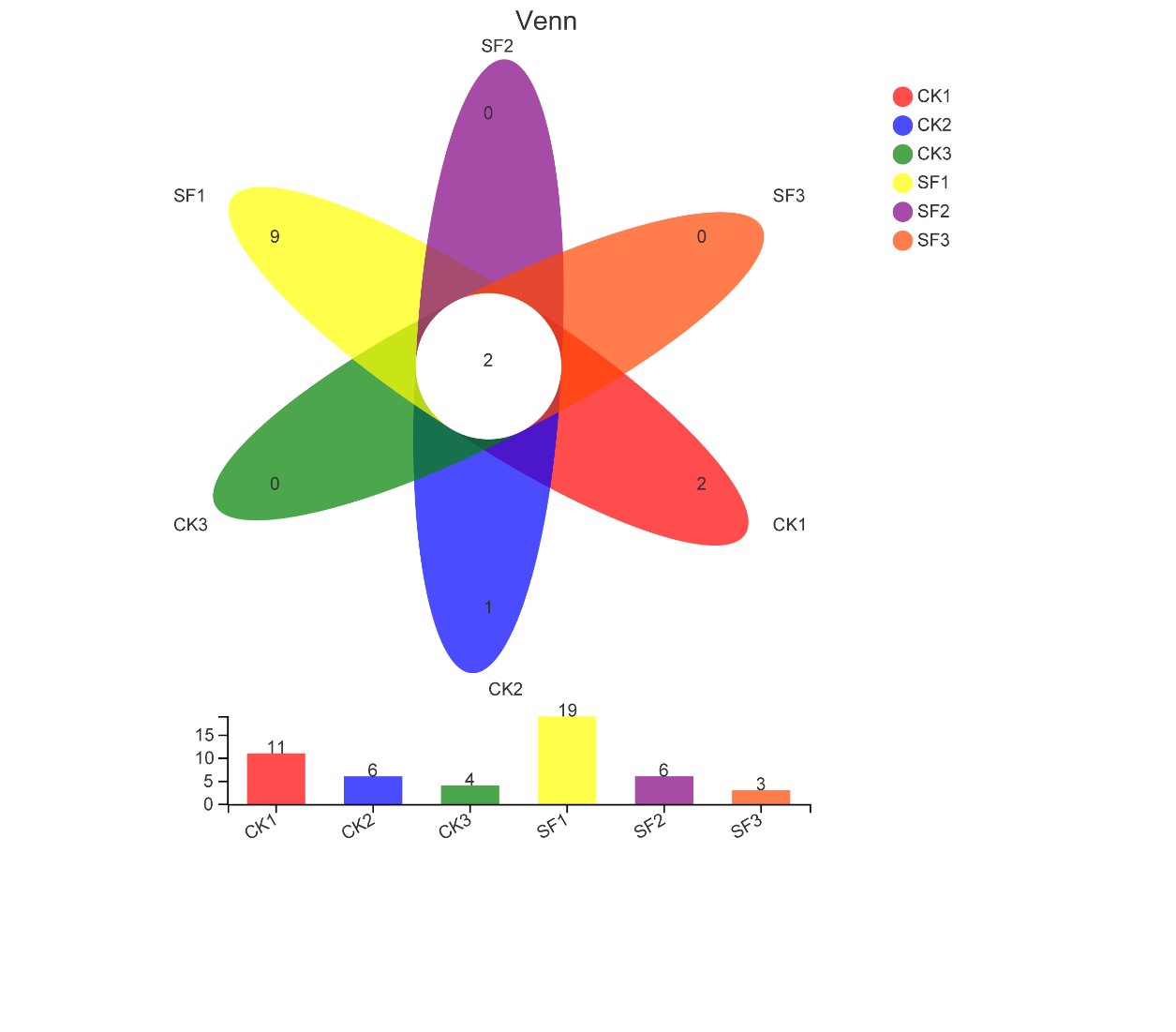


**Figure S1.** Venn plots of microorganisms (OTU levels) in BH samples at different fermentation stages. In the petal map is the number of species unique to the corresponding group, and the center is the number of species common to all groups (above). The figure below is a histogram of the total number of species in each group at the OTU level.
